# Evaluation of Vitamin D Levels in Pediatric Patients With Recurrent Aphthous Stomatitis

**DOI:** 10.7759/cureus.32064

**Published:** 2022-11-30

**Authors:** Fatmagül Başarslan, İlknur Kaba

**Affiliations:** 1 Pediatrics, Hitit University, Çorum, TUR

**Keywords:** pediatric nutrition, vitamin-d, children, recurrent aphthous stomatitis, oral diseases

## Abstract

Background: Recurrent aphthous stomatitis is one of the most common oral mucosal diseases. It is characterized by recurrent painful attacks. Its etiology is unknown. Vitamin D (vit D) is a steroid vitamin with immunomodulatory and anti-inflammatory effects. It is thought that oral cavity diseases may occur in vitamin D deficiency. This study aimed to investigate vit D levels in pediatric patients with recurrent aphthous stomatitis.

Methods: In this retrospective study, 86 children with recurrent aphthous stomatitis and 71 age-matched healthy children were included in the study. The 25-hydroxyvitamin D levels examined with the enzyme immune assay were recorded for both groups.

Results: Serum vit D level was 12±4.5 ng/ml in the group with aphthous stomatitis and 31±7 ng/ml in the healthy group. A statistically significant difference was found in vit D levels between the two groups (p<0.001).

Conclusions: Vit D levels were significantly low in children with recurrent aphthous stomatitis. Our findings suggest that low vit D levels may be associated with recurrent aphthous stomatitis.

## Introduction

Recurrent aphthous stomatitis is one of the most common oral mucosal diseases. These lesions are characterized by recurrent painful attacks and can be seen as single or multiple painful ulcers on the oral mucosa, cheek, lip, and tongue [1,2]. It can rarely be seen on the palate and gingival mucosa. Eruptions have an erythematous halo around them and are covered with a fibrous layer [1,2]. Aphthous stomatitis affects approximately 25% of the general population [3]. The incidence in children in North America is 1.1% [4]. The etiology of recurrent aphthous stomatitis is unknown. Genetic predisposition, viral and bacterial infections, food allergies, systemic diseases (such as Crohn's, ulcerative colitis, and celiac disease), local trauma, stress, poor oral hygiene, changes in oral flora, and vitamin and mineral deficiencies may be triggers [1]. In current studies, vitamin deficiencies are investigated in the etiology of recurrent aphthous stomatitis. Several studies showed that patients with recurrent aphthous stomatitis had deficiencies of iron, B1, B2, and B6 vitamins [5,6].

Vit D is a fat-soluble, steroidal vitamin and plays an important role in calcium metabolism. Recent studies have focused on the immune regulatory role of vit D due to the high abundance of vit D receptors in antigen-presenting cells, macrophages, dendritic cells, and T cells [7,8]. Vit D stimulates the innate immune response by increasing the differentiation of monocytes and the chemotactic and phagocytic effects of macrophages [8,9]. Previous studies have shown that vit D deficiency significantly increases the risk of developing autoimmune diseases (such as systemic lupus erythematosus, rheumatoid arthritis, asthma, inflammatory bowel diseases, multiple sclerosis, and type 1 diabetes) [10-12].

There are limited publications in the literature on the effect of vit D on oral cavity health. Studies investigating the relationship between vit D and aphthous stomatitis were mostly conducted in adults and very few studies are focused on children. This study aimed to evaluate the level of vit D in recurrent aphthous stomatitis in the pediatric population.

## Materials and methods

Patients who were diagnosed with recurrent aphthous stomatitis and followed up in the pediatric health and diseases outpatient clinic of Hitit University Training and Research Hospital, Çorum, Turkey, between January 2020 and August 2022 were included in this retrospective study. Patients who applied with the complaint of sores in the mouth other than aphthous stomatitis but were not diagnosed with aphthous stomatitis, who were not between the ages of one to 18, and who had incomplete examinations and tests, were not included in the study. The clinical characteristics of the patients and the measured 25-hydroxyvitamin D levels were evaluated retrospectively. Eighty-six children aged one to 18 years in both gender groups were included in the study, and 71 healthy children in the same age group were included as the control group. Telephone interviews were conducted with the patients. It was found that all patients had at least two minor attacks. The control group consisted of healthy children without any complaints and systemic diseases who came for routine control. Those under the age of one, patients with chronic diseases, those who received vit D supplements in the last six months, patients with major herpetiform aphthous stomatitis, and those who had a single episode of aphthous stomatitis were excluded from the study. Age, gender, and vit D levels were recorded. A 25-hydroxyvitamin D level of 30 ng/ml was evaluated as sufficient, 20 to 30 ng/ml as insufficient, and below 20 ng/ml as a deficiency. The ethics committee approval of the study was obtained from the Ethics Committee of Hitit University Training and Research Hospital.

Statistical analysis

Statistical analysis of the research data was performed using the SPSS version 22.0 (IBM Corp., Armonk, NY, USA). Descriptive statistics of categorical variables were presented as numbers and percentages (%). Descriptive statistics for continuous variables were presented as mean ± standard deviation and median ± interquartile range (IQR) according to data distribution. The conformity of data to normal distribution was tested using Kolmogorov-Smirnov and Shapiro-Wilk tests. In the comparison of numerical variables between two independent groups, the t-test (Students' t-test) was used in independent groups if the data were normally distributed, and the Mann-Whitney U test was used if the data were not normally distributed. A chi-square test or Fisher's exact test was used based on the number of data in the crosstab cells for ratio or relationship comparisons of categorical variables. The statistical significance level was accepted as p<0.05.

## Results

**Figure 1:**
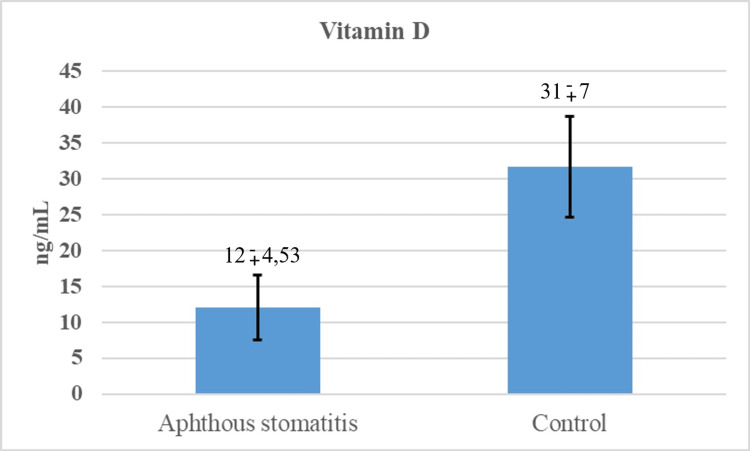
The mean serum vitamin D concentrations of aphthous stomatitis and control groups

In our study, the data of a total of 157 patients were analyzed. Of the patients, 86 (54.8%) were in the aphthous stomatitis group and 71 (45.2%) were in the control group. Around 51.6% (n=81) of the patients were male and 48.4% (n=76) were female. There were no significant differences between study groups according to gender distribution (P=0.280). The mean age of the patients was 8.36±4.28. The mean age of the patients in the aphthous stomatitis group was 8.61±4.22 and the mean age of the patients in the control group was 8.06±4.36. The mean age of the patients was not statistically different between the study groups (P=0.423; Table 1). Vit D was statistically significantly different between the study groups (P<0.001; Table 1). The results are shown in Figure 1.

**Table 1:** Comparison of gender, age, and vitamin D values between the study groups

		Aphthous stomatitis	Control	P-value
		(n=86)	(n=71)	
Gender	Male	41 (47.7%)	40 (56.3%)	0.280
	Female	45 (52.3%)	31 (43.7%)	
Age		8.61±4.22	8.06±4.36	0.423
Vitamin D		12±4.53	31±7	<0.001

## Discussion

Recurrent aphthous stomatitis is one of the most common oral mucosal diseases. Although its etiology is not fully known, it is known that vitamin and mineral deficiencies might be triggers [1,3,5,6]. In this study, we aimed to evaluate the vit D levels in children with recurrent aphthous stomatitis and to investigate the potential effect of vit D in its etiology. In their study of Öztekin et al. and Nalbantoğlu et al. found that vit D levels were significantly lower in the patient groups when serum vit D levels were compared in healthy individuals and individuals with recurrent aphthous stomatitis [2,13]. Based on these data, vit D supplementation has been recommended in patients with recurrent aphthous stomatitis [2,13]. In another study, it was shown that topical oral gel with vit D reduced oral mucositis after radiotherapy [14]. In our study, we found low vit D levels in children with recurrent aphthous stomatitis (as seen above in Figure 1).

In recurrent aphthous stomatitis pathology, the presence of chronic inflammation suggests an immunological basis. T-cell immunity plays an important role in the development of recurrent aphthous stomatitis. Accordingly, cluster of differentiation (CD)4 T lymphocytes are more dominant in the early stage of aphthous stomatitis, while CD8 T lymphocytes are dominant in the ulcerative stage [15]. It has been shown that there is an increase in T helper 1 proinflammatory cytokines and a decrease in T helper 2 anti-inflammatory cytokines in the pathogenesis of recurrent aphthous stomatitis [16,17].

The two basic forms of vit D are ergocalciferol, which is vitamin D2, and cholecalciferol, which is vitamin D3, and both forms are inactive. After hydroxylation in the liver and kidney, it forms the active form with 1,25 dihydroxy-cholecalciferol [18]. The active form of vit D exerts its effect by binding to nuclear receptors, as in other steroid hormones [8]. Normal serum vit D (25-hydroxy) concentration is 30 to 50ng/ml. A level of 21 to 29ng/ml is defined as insufficiency and a level of below 20ng/ml is deficiency [19]. In our study, levels of 25-hydroxy vit D were examined, and vit D deficiency was detected in the recurrent aphthous stomatitis group.

It has been shown that vit D plays a role in immune processes, has anti-inflammatory and antimicrobial effects, inhibits cell proliferation, and stimulates differentiation [20]. Vit D stimulates the innate immune response by increasing the differentiation of monocytes and the chemotactic and phagocytic effects of macrophages. It is emphasized that the effects of vit D on cell proliferation and differentiation can significantly affect oral cavity health [20]. It has been shown that antimicrobial defensin and cathelicidin are synthesized through vit D receptors and have an antibacterial effect against oral pathogens by providing a nonspecific immune response [21]. Previous studies have shown that vit D decreases the release of T helper 1 type cytokines, increases the release of T helper 2 type cytokines, and stimulates the formation of T regulatory lymphocytes by inhibiting the differentiation and maturation of dendritic cells [8,9]. The important role of vit D in the innate and acquired immune system is its ability to influence the synthesis of proinflammatory cytokines. And the presence of vit D receptors on macrophages, dendritic cells, and T- and B-lymphocytes may explain its potential relationship with recurrent aphthous stomatitis.

In Behçet's disease and periodic fever, aphthous stomatitis, pharyngitis, and adenitis (PFAPA) syndrome, which are diseases associated with recurrent aphthous stomatitis, Stagi et al. showed that vit D was significantly lower in children with PFAPA syndrome and that when 400 IU vit D was administered daily, there was a decrease in fever episodes and frequency [22,23]. The study by Bahramian et al. compared individuals with recurrent aphthous stomatitis and healthy individuals and found significantly lower serum vit D levels in the aphthous stomatitis group [24]. Vit D levels were also found to be low in studies conducted with patients with periodontitis [21,25]. In our study, we found that vit D levels were significantly lower in children who had recurrent aphthous stomatitis attacks. Krawiecka et al. found no difference in vit D levels between individuals with aphthous stomatitis and healthy individuals [17]. It has been found in the literature that studies investigating the relationship between recurrent aphthous stomatitis and vit D were mostly conducted with adults [2,5,17].

## Conclusions

Our study is one of the rare studies conducted in children with recurrent aphthous stomatitis. There are studies conducted in the adult population with recurrent aphthous stomatitis. The number of studies on the pediatric population should be increased. We think that this study has limitations due to its retrospective nature. Prospective studies with larger groups are recommended to examine the role of vit D in the etiology of recurrent aphthous stomatitis. In addition, it is essential to evaluate the potential therapeutic and protective role of vit D in recurrent aphthous stomatitis.
